# Management of a tibial periprosthetic fracture following revision knee arthroplasty using a pulsed electromagnetic field stimulation device: a case report

**DOI:** 10.4076/1757-1626-2-8706

**Published:** 2009-08-05

**Authors:** Ashtin Doorgakant, Mohammed A Bhutta, Hans Marynissen

**Affiliations:** Trauma and Orthopaedics, North Western Deanery, East Lancashire Hospital Trust13 Cringle Drive, Cheadle, Cheshire, SK8 1JHUK

## Abstract

Periprosthetic fractures associated with total knee arthroplasty are rare but present a challenging problem particularly when associated with revision arthroplasty. Fractures around tibial stems are particularly difficult with no accepted technique in their management. This case describes a tibial periprosthetic fracture following a revision knee arthroplasty which was successfully managed with a Pulsed ElectroMagnetic Field bone stimulation device. We believe this to be first reported use of a bone stimulation device in this clinical environment.

## Case presentation

**Figure 1.**
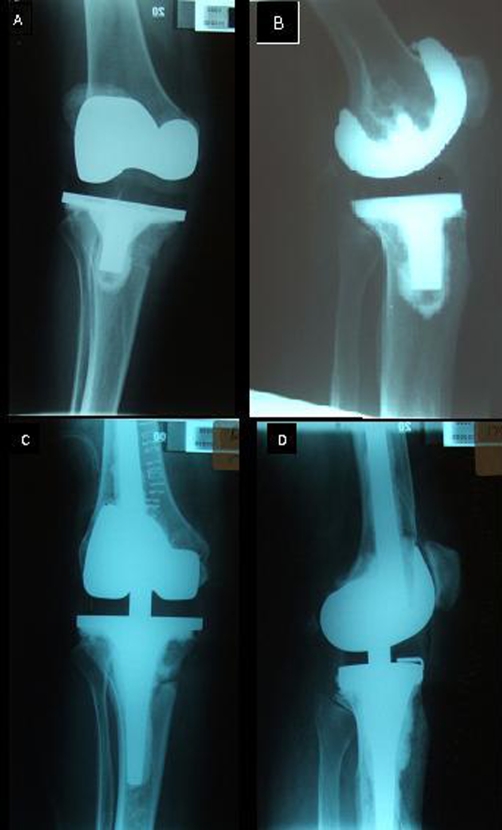
Immediate AP and Lateral of Primary total knee arthroplasty post MCL reconstruction **(A & B)** and immediate postoperative films **(C & D)**.

A 75 year old Caucasian lady suffering from osteoarthritis of her right knee with 20 degrees of valgus deformity proceeded to a total knee replacement (Press Fit Condylar: Cruciate retaining: Depuy, Leeds, Yorkshire, UK). Twelve months later the valgus deformity reoccurred with loss of function of the medial collateral ligament (MCL). The MCL was reconstructed using Leeds-Keio connective tissue prosthesis and biotenodesis screw (Figure 1A & 1B). However, this failed 6 months from reconstruction and a revision total knee arthroplasty was undertaken with a hinged short stem cemented prosthesis (Endoplus UK).

A radiograph taken immediately post-operatively demonstrated a periprosthetic fracture at the level of the tibial stem on the medial side (Figure 1C & 1D). This was attributed to the site of the biotenodesis screw from the previous MCL repair. The tibial stem bypassed the fracture and therefore routine rehabilitation was continued.

**Figure 2.**
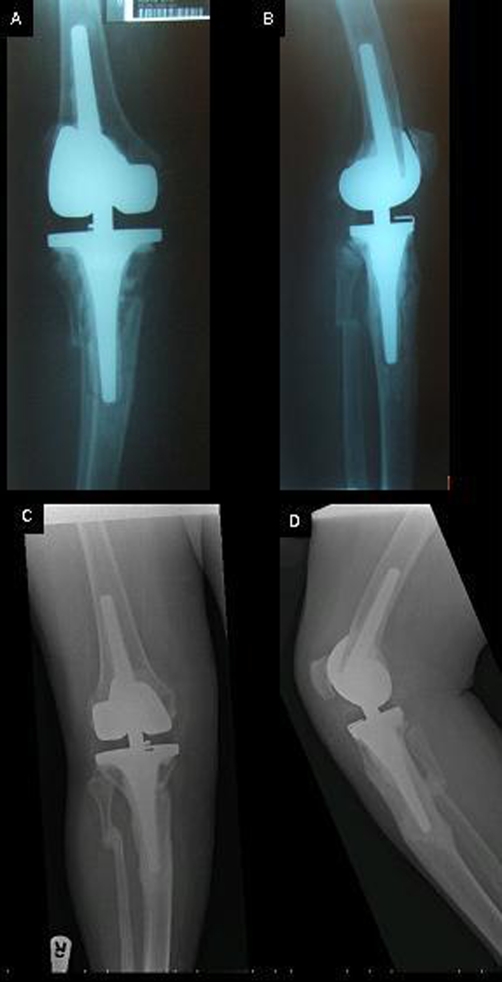
AP and Lateral of Periprosthetic fracture **(A & B)** and at union **(C & D)**.

Two months later the patient described increasing pain in the operated limb, with radiographs demonstrating extension of the fracture to the lateral cortex but with no displacement. Therefore, a non weight-bearing long-leg light cast was applied, unfortunately a further fall two weeks later fractured the fibula producing a valgus deformity (Figure 2A & 2B).

The tibial prosthesis appeared well fixed proximally and therefore non-operative management was initiated with Manipulation under Anaesthesia (MUA) and long-leg plaster of paris (POP). The fracture remained mal-aligned therefore a further MUA and POP was performed and a Pulsed Electromagnetic Field Device (PEMFD) (Physio-stim, Intavent ORTHOFIX Ltd, Maidenhead, UK) was applied. The device was used for at least 3 hours per day until the fracture healed.

At 4 weeks from application the fracture was assessed under anaesthesia and found to have some stability and converted from a long leg POP to a hinged knee brace. The patient was allowed to increase weight-bearing to 50% of body-weight converting to full weight-bearing 4 months from the injury. The fracture was monitored with serial radiographs, clinical examination in the outpatient department and theatre to assess fracture healing and ensure acceptable limb alignment.

Eight months from sustaining the periprosthetic fracture and 7 months from the application of the PEMFD complete bony union was achieved clinically and radiologically (Figure 2C & 2D). At 21 months from fracture and 14 month from bony union the patient is mobilising fully weight-bearing and is asymptomatic.

## Discussion

Total knee arthroplasty (TKA) surgery is performed in ever increasing numbers. One potential complication is of periprosthetic fractures during primary and revision surgery or a result of trauma [1]. Periprosthetic fractures of TKA are most common around the femur with an incidence of 0.3-2.5% in primary surgery and 1.6-38% in revision surgery [1]. Fractures involving the tibia are less common with an incidence of 0.1% and 0.4% in primary and revision surgery respectively [2].

The management of tibial periprosthetic fractures is a challenging problem, attributed to its rarity and patient population with poor bone stock and healing. Tibial periprosthetic fractures have been classified by Felix et al. based upon anatomical location (Types I-IV), fixation of implants (A: well-fixed or B: loose) or timing of the fracture (C: intra-operative) [2]. Possible treatment strategies have been suggested dependent upon this classification [2-7] (Table 1).

**Table 1.** Fracture Classification and Potential Management Options

Fracture Type		Possible Management
I Plateau	A (Fixed Prosthesis)	Non-operative
	B (Loose Prosthesis)	Revision surgery: long-stemmed, modular components, bone graft.^2-5^
II Adjacent to Stem	A	Standard fracture Management principles
	B	Revision surgery: use of bone graft^3,6^
III Distal to Stem	A	Standard fracture Management principles
	B	Proximal: Longer stemmed component Distal: ORIF and delayed revision^3,4^
IV Tibial Tubercle	A	Standard fracture Management principles

Type-II fractures occur adjacent to the tibial stem of which Type-A represent a well fixed implant as in this case (given it was noted postoperatively and the lack of cement extrusion in the immediate post-operative films). However, in-keeping with the literature the patient sustained this fracture following a mild traumatic event, which is commonly seen with modern condylar knee designs [2-3] and was possibly contributed to by the stress riser at the site of MCL reconstruction and disuse osteopenia [1,8].

Displaced Type-IIA fractures present a particularly challenging management problem. Those that remain undisplaced can be treated non-operatively. However, if displaced open reduction internal fixation (ORIF) techniques are employed to achieve correct alignment and stability [2,3,6]. In this case a very proximal medial tibial fracture extending just beyond the tibial stem would compromise fracture fixation with inadequate screw purchase. Ordinarily, if this cannot be achieved then revision surgery is indicated, but can create extensive bone deficits from both tibial plateaus during the removal of the well fixed tibial component [3]. Considering these technical difficulties, previous surgeries and patient requirements the patient was managed non-operatively with the use of closed reduction and casting. In isolation, with the fracture pattern and patient biology the probability of non-union or delayed union was high. To address this, a PEMFD was applied.

Current bone stimulating devices fall into two broad technologies that of ultrasound and electric/electromagnetic [9]. Low-intensity pulsed ultrasound devices have shown clinical benefit in non-unions, although concerns for its use in the presence of metallic implants have been refuted [9,10]. Their application requires a window to be made in the cast directly over the fracture site and the use of a gel to transmit the ultrasound. Electrical devices can be invasive or non invasive and use differing techniques to produce the healing/maturing effect upon fracture tissue. Non-invasive PEMF devices are also applied at the fracture site but can be applied external to casts prevent cast weakness and loss of fracture reduction [11].

The mechanism of action remains under investigation but evidence suggests PEMFD regulate proteoglycan and collagen synthesis and increase bone formation in models of endochondral ossification, encouraging mineralisation and angiogenesis, increasing DNA synthesis and altering the cellular calcium content in osteoblasts to produce results equivalent to bone grafts in clinical studies with union in approximately 80% of cases achieved between 14 and 21 weeks [9-13]. The PEMFD is licensed for fracture gaps less than 10 mm, in the absence of pseudoarthrosis or pathological fracture and for the treatment of non-union. It must be applied for 3 hours per day for a minimum of 180 consecutive days to achieve complete bony union [14].

In this case complete bony union required 7 months from application which is 1 month longer than that expected by the manufacturers. However, given the complexity of the fracture this still represents a successful outcome. There is only one other case report in the literature describing the use of capacitively coupled electromagnetic field in the management of a Type-IIIA (distal to tibial stem) periprosthetic fracture. However, our case represents a much more complex fracture pattern involving the tibial metaphysic and fibula in which there was a fracture gap greater than 10 mm and the PEMFD was applied immediately.

This is the first report in the literature of the successful use of a PEMFD for Type-IIA tibial periprosthetic fractures. Although there are limitations of a single case, the successful application of the PEMFD in this environment could suggest broadening the scope of indications of such devices and increase the armamentarium available to deal with such injuries, in an increasingly frail population.

## Abbreviations

MCL: Medial collateral ligament
MUA: Manipulation under anaesthesia
ORIF: Open reduction internal fixation
PEMFD: Pulsed Electromagnetic Field Device
POP: Plaster of paris
TKA: Total knee arthroplasty
